# Low-Cost Homemade Simulation Trainer for the Erector Spinae Plane Block

**DOI:** 10.7759/cureus.111869

**Published:** 2026-07-01

**Authors:** Philip S Fallah, Joshua Perese, Benjamin Liotta, Jessica Oswald

**Affiliations:** 1 Department of Emergency Medicine, University of California San Diego, San Diego, USA; 2 Department of Anesthesiology and Center for Pain Management, University of California San Diego, San Diego, USA

**Keywords:** erector spinae plane (esp) block, erector spinae plane model, low-cost simulation module creation, low-cost ultrasound model, ultrasound gel model trainer

## Abstract

Introduction

The ultrasound-guided erector spinae plane block (USG-ESPB) is an interfascial plane block that provides analgesia for several conditions causing truncal pain. Many training models are available for purchase but may be cost-prohibitive for anesthesia and emergency medicine training programs. This article reports on a low-cost, reusable gel thoracic spine phantom for teaching the USG-ESPB.

Materials and methods

The phantom was made using an Axis Scientific skeleton model modified to include only the thoracic vertebrae, a plastic bin, and Huminic medical gel #0. The spine model was suspended in the bin, and the gel was melted at 275ºF and poured over it. Eight board-certified pain physicians and regional anesthesiologists at a pain management fellowship site were then surveyed to assess the characteristics of the phantom by touch and ultrasound.

Results

Of the participating physicians, 7/8 (87.5%) agreed or strongly agreed that the phantom reproduced the texture and resistance of human tissue. All participants (8/8, 100%) agreed or strongly agreed that the gel medium provided sufficient ultrasound penetration and enabled them to identify targets within it. All participants (8/8, 100%) also agreed or strongly agreed that the phantom was more transportable than commercial models and cadavers. Additionally, 7/8 (87.5%) agreed or strongly agreed that the phantom was more reproducible than commercial models, while 6/8 (75%) agreed or strongly agreed that it was more reproducible than a cadaver.

Conclusions

These findings highlight the potential for low-cost gel models to enhance regional anesthesia education. This phantom’s favorable ratings in tissue realism, ultrasound visibility, portability, reproducibility, and reusability, especially when compared to commercial models and cadaver labs, support its value as an educational tool. Similar models may expand access to procedural training without the prohibitive cost of commercial simulators or cadavers.

## Introduction

The ultrasound-guided erector spinae plane block (USG-ESPB) is a large-volume interfascial plane block that provides analgesia for several acute conditions causing somatic truncal pain, including rib and lumbar transverse process fractures, burns, and acute zoster [1-4]. Additionally, there are case reports and case series reporting effectiveness for visceral pain in renal colic, acute pancreatitis, and cancer-related pain [2,5]. It is an easy-to-learn, safe, and efficacious regional analgesic procedure, first described in the anesthesia literature [6]. As ultrasound and regional anesthesia are becoming increasingly integrated in emergency medicine and anesthesia training curricula, the ESPB is increasingly recognized as an effective adjunct and alternative to opioids [2,6]. To date, 57% of emergency medicine training programs have a dedicated regional anesthesia curriculum, including didactic sessions (67%), online resources (54%), and supervised training with real patients (48%). The most commonly taught blocks include the forearm (74%), femoral (69%), and posterior tibial (43%) nerve blocks [7].

Based on the medical education literature, a combination of didactic sessions, teaching sonoanatomy on healthy living individuals, and needle-tracking practice on training phantoms is a common approach to teaching ultrasound-guided regional anesthesia (e.g., fascia iliaca) to physician trainees [8-10]. Many training phantoms are available for purchase and modification. To date, one study has described a low-cost gelatin phantom for ultrasound-guided lumbosacral training to anesthesia trainees [11]. Other low-cost phantoms use 3D printing, which may also be cost-prohibitive for many training programs [12,13]. Thus, there exists a growing need for a cost-effective spine model to teach trainees how to perform an ESPB. This pilot feasibility study describes a low-cost, reusable gelatin thoracic spine phantom to teach the USG-ESPB.

An abstract of this manuscript was presented at the 2026 AAPM Annual PainConnect conference on March 6th, 2026.

## Materials and methods

**Figure 1:**
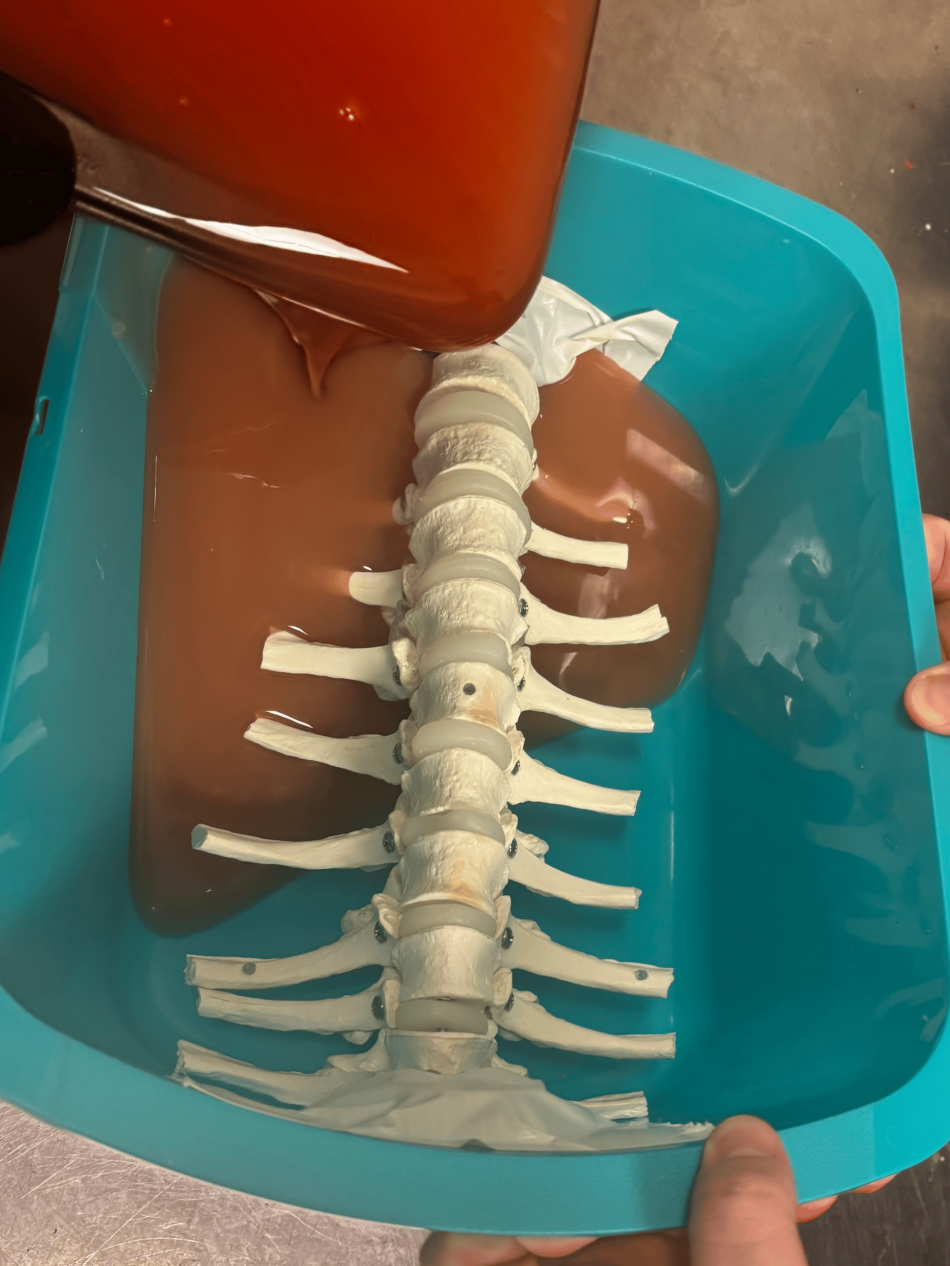
Melted Humimic SimuGel, colored with various lipstick shades, poured over the thoracic spine model

This phantom was made using the thoracic spine of an Axis Scientific skeleton model (Axis Scientific, Anatomy Warehouse, USA), a plastic bin measuring 14 x 8.5 x 6 inches, and Humimic SimuGel #0 (Humimic Medical, Greenville, SC, USA). The plastic skeleton model was modified to remove the cervical spine, lumbar spine, lateral ribs, and pelvis. Four thoracic vertebrae were removed to fit inside the plastic bin. The remaining nine thoracic vertebrae (T4-T12) were suspended in the bin by taping the ends of the metal rod to the inner walls, with the spinous processes positioned approximately 2 cm above the base of the bin. The Humimic SimuGel was melted at 275ºF and poured onto the entire spine (Figure 1). The ends of various colored lipsticks were added to the gel to provide color and opacity, hiding bony targets in the otherwise clear gel medium. The gel could be reused after multiple needle passages by removing it, melting it, and repouring it into the mold over the spine.

Assessment of usefulness

**Figure 2:**
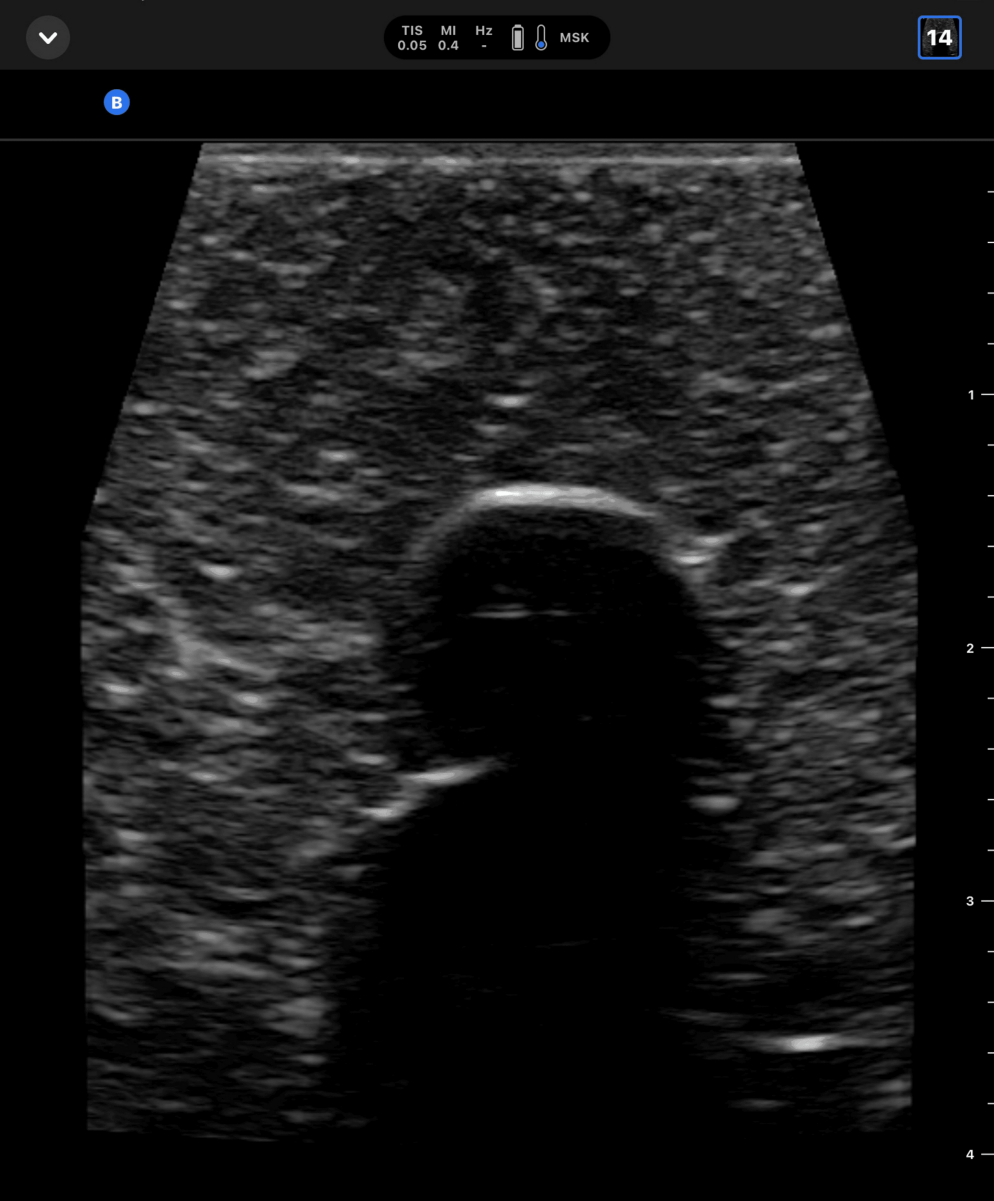
Transverse process of T8 using the linear Butterfly iQ3 probe

Eight regional anesthesiologists and board-certified pain physicians who have performed over 100 erector spinae blocks at a single pain management fellowship-training site were asked to evaluate the phantom model by palpation and ultrasonography. A linear Butterfly iQ3 probe (Butterfly Network, Inc., Burlington, MA, USA) attached to a first-generation 11-inch iPad Pro (Apple Inc., Cupertino, CA, USA) was provided for sonographic assessment, which faculty members used at their discretion (Figure 2). They then completed a survey to assess the phantom's characteristics and their comparison with commercial models and cadavers (see Appendix A). While commercial models were not available for direct comparison, all participants had previously used them for various blocks during their training. Using a four-point Likert scale ranging from 1 (strongly disagree) to 4 (strongly agree), participants rated seven statements assessing the phantom. The survey was based on a literature review of ultrasound-guided regional anesthesia simulators [12]. The survey was designed to obtain expert impressions of characteristics considered important for procedural simulation training as an initial assessment of the phantom's potential educational utility.

## Results

As shown in Table 1, 87.5% of participating physicians agreed or strongly agreed that the phantom reproduced the texture and resistance of human tissue (average score 3, SD ±0.92). 100% of participants agreed or strongly agreed that the gel medium had sufficient ultrasound penetration (average 3.75, SD ±0.46) and that they could identify targets within the gel medium (average 3.5, SD ±0.53). 100% of participants agreed or strongly agreed that the phantom was more transportable than commercial models (average 3.75, SD ±0.46). 100% strongly agreed that it was more transportable than a cadaver (average 4, SD ±0). 87.5% agreed or strongly agreed that the phantom was more reproducible than commercial models (average 3.25, SD ±1.04). 75% agreed or strongly agreed that it was more reproducible than a cadaver (average 3.38, SD ±1.19).

**Table 1:** Individual responses to the survey in Appendix A with mean and standard deviation for each question 1 = strongly disagree, 2 = disagree, 3 = agree, 4 = strongly agree

Years since completing fellowship	Q1: Today’s phantom reproduced the texture and resistance of human tissue	Q2: Today’s phantom reproduced had sufficient ultrasound penetration to enable identification and location of targets	Q3: Today’s phantom had targets that were clearly distinguished from the surrounding medium on ultrasound	Q4: Today’s phantom would be easily transportable compared to a COMMERCIAL MODEL	Q5: Today’s phantom would be easily transportable compared to a CADAVER	Q6: Today’s phantom would be easily reproducible compared to a COMMERCIAL MODEL	Q7: Today’s phantom would be easily reproducible compared to a CADAVER
0-5	3	4	3	4	4	3	4
0-5	3	4	3	4	4	4	4
15+	1	3	3	3	4	1	1
6-10	4	4	4	4	4	4	4
0-5	4	4	4	4	4	3	2
0-5	3	3	3	4	4	4	4
6-10	3	4	4	3	4	3	4
0-5	3	4	4	4	4	4	4
Average Scores	3.00	3.75	3.50	3.75	4.00	3.25	3.38
STD	0.93	0.46	0.53	0.46	0.00	1.04	1.19

## Discussion

This gel phantom is an easy-to-make, relatively low-cost, transportable model that both anesthesia and emergency medicine training programs can use to teach ESPB to their trainees. For example, the BlockSim SKU:BLK (NASCO Healthcare, 2020) weighs 55 pounds and costs over $33,000 [14]. Other, more cost-effective models reported in the literature utilize 3D printing, which requires equipment not all training programs have access to [12]. The phantom model described in this report can be made with readily available equipment and cost just over $600 in materials alone. The gel itself accounted for about 60% of the cost but is essentially a single purchase and can be melted and reused.

The physicians surveyed in this study agreed that the phantom reproduced the texture and resistance of human tissue and had sufficient ultrasound penetration to identify bony targets within the gel medium. This is an important feature for any ESPB model to demonstrate, as the transverse process is an important safety landmark to avoid accidental intrathoracic or retroperitoneal puncture. Furthermore, the participants perceived the model as more transportable and reproducible than commercial models and cadavers, based on their prior experiences with these training modalities. Additionally, unlike other common models, the gel can be melted and remade after its integrity is compromised by multiple needle punctures [15].

While this study only describes a thoracic spine model, it could be recreated to include a thoracolumbar spine model. This would require using either the same spine model with different modifications or a different spine model. Doing so would provide trainees with practice differentiating the thoracic spine and its lateral ribs from the lumbar spine. The ease with which the gel can be retooled for different models highlights its versatility and value.

These findings suggest that low-cost, reproducible gel models may serve as a feasible adjunct to regional anesthesia education, particularly in settings where access to cadavers or expensive equipment is limited. By reducing financial and logistical barriers to simulation-based training, this model may improve access to ESPB instruction.

This study has several limitations. The phantom was evaluated by a small sample of eight experts from a single institution using an unvalidated, subjective survey instrument. Therefore, the findings do not determine whether the model improves learner confidence, procedural competency, or clinical performance. Additionally, commercial phantoms and cadavers were not available for direct comparison; thus, perceptions of portability and reproducibility reflect participants' opinions based on prior experience rather than formal comparative evaluation. Finally, participants did not perform needle insertion during their evaluation; therefore, feedback was limited to the phantom's tactile characteristics and sonographic appearance. Future studies should include needle insertion, larger cohorts, and objective educational outcomes such as learner confidence, procedural competency, and clinical performance.

## Conclusions

This affordable, reusable gelatin phantom may offer an accessible solution for teaching USG-ESPB in emergency and pain medicine training programs. Participants reported favorable impressions regarding tissue realism, ultrasound visibility, portability, reproducibility, and reusability, supporting its potential value as a training adjunct. As ultrasound-guided regional anesthesia becomes increasingly integrated into training curricula, models like this can help expand access to hands-on procedural training without the prohibitive costs of commercial simulators or the logistical challenges of cadaver labs. Additional studies are needed to evaluate its educational effectiveness and role within emergency and pain medicine curricula.

## Appendices

Phantom utility survey

Years since completing fellowship: 0-5 ___ , 6-10 ___, 11-15 ___ , 15+ ___

On a scale of 1-4 (1 = strongly disagree, 2 = disagree, 3 = agree, 4 = strongly agree), please rate the following statements with regard to the ESPB in the thoracic region:

1. Today’s phantom reproduced the texture and resistance of human tissue.

2. Today’s phantom reproduced had sufficient ultrasound penetration to enable identification and location of targets.

3. Today’s phantom had targets that were clearly distinguished from the surrounding medium on ultrasound

4. Today’s phantom would be easily transportable compared to a commercial phantom

5. Today’s phantom would be easily transportable compared to a cadaver

6. Today’s phantom would be easily reproducible compared to a commercial model

7. Today’s phantom would be easily reproducible compared to a cadaver
